# The effect of caffeic acid phenethyl ester on the functions of human monocyte-derived dendritic cells

**DOI:** 10.1186/1471-2172-10-39

**Published:** 2009-07-16

**Authors:** Li-Chieh Wang, Yu-Li Lin, Yu-Chih Liang, Yao-Hsu Yang, Jyh-Hong Lee, Hsin-Hui Yu, Wen-Mein Wu, Bor-Luen Chiang

**Affiliations:** 1 Department of Pediatrics, National Taiwan University Hospital, 7 Chung-Shan South Rd, Taipei 100, Taiwan, Republic of China; 2 Department of Medical Research, National Taiwan University Hospital, 7 Chung-Shan South Rd, Taipei 100, Taiwan, Republic of China; 3 School of Medical Laboratory Science & Biotechnology, College of Medicine, Taipei Medical University, 250 Wu-Xin Street, Taipei 110, Taiwan, Republic of China; 4 Department of Nutritional Science, Fu-Jen Catholic University, 510 Chung-Cheng Rd, Hsinchuang, Taipei County 24205, Taiwan, Republic of China

## Abstract

**Background:**

Propolis, an ancient herbal medicine, has been reported the beneficial effect both in asthma patients and murine model of asthma, but the mechanism was not clearly understood. In this study, the effect of caffeic acid phenethyl ester (CAPE), the most extensively studied components in propolis, on the functions of human monocyte-derived dendritic cells (MoDCs) was investigated.

**Results:**

CAPE significantly inhibited IL-12 p40, IL-12 p70, IL-10 protein expression in mature healthy human MoDCs stimulated by lipopolysaccharides (LPS) and IL-12 p40, IL-10, IP-10 stimulated by crude mite extract. CAPE significantly inhibited IL-10 and IP-10 but not IL-12 expression in allergic patients' MoDCs stimulated by crude mite extract. In contrast, the upregulation of costimulatory molecules in mature MoDCs was not suppressed by CAPE. Further, the antigen presenting ability of DCs was not inhibited by CAPE. CAPE inhibited IκBα phosphorylation and NF-κB activation but not mitogen-activated protein kinase (MAPK) family phosphorylation in human MoDCs.

**Conclusion:**

These results indicated that CAPE inhibited cytokine and chemokine production by MoDCs which might be related to the NF-κB signaling pathway. This study provided a new insight into the mechanism of CAPE in immune response and the rationale for propolis in the treatment of asthma and other allergic disorders.

## Background

Asthma is the leading chronic disease in children. Recent advance of asthma medication has decreased the mortality and morbidity of asthma. Among them, inhaled corticosteroid is the mainstay treatment. Other medications such as theophylline, long-acting beta_2_-adrenergic agonist, leukotriene modifier and anti-IgE treatment, alone or in combination of inhaled corticosteroid, have increased the control of asthma. However, these drugs do have some side effects such as oral thrush, inhibition of growth[1] or increased risk of severe asthma exacerbation [2]. Difficult-to-treat asthma patients do still have persistent symptoms even with the best therapy [3]. Therefore, several other approaches to asthma treatment are extensively studied. The subcutaneous immunotherapy has some effect but the anaphylactic side effect and frequent injection limit the application [4]. Sublingual immunotherapy, which was safe in children, has only low to moderate clinical efficacy in mild to moderate persistent asthma [5,6]. Other novel treatments in murine model of asthma, such as central deoxycytidyl-deoxyguanosine (CpG) dinucleotide inhalation, DNA vaccination and antisense oligonucleotide are not yet proved in human [7].

Propolis, the natural resinous products collected by honeybees from various plant sources, is well known for the management of respiratory problems in herbal medicine [8]. In clinical studies, Khayyal *et al*. administered the aqueous extract of propolis to patients with mild to moderate asthma daily for 2 months in combination with oral theophylline [9]. They found that the number of nocturnal asthma attacks decreased significantly in the propolis treatment group compared to the placebo group. In addition, the lung function of patients treated with propolis improved after 2 months while the placebo group did not. Finally, the sera of patients in the propolis group had significantly lower levels of tumor necrosis factor (TNF)-α, intercellular adhesion molecule (ICAM)-1, interleukin (IL)-6, IL-8, leukotrienes and prostaglandin (PG)E_2 _after the 2 months treatment period, but these changes were not identified in the placebo group. In murine model of asthma, propolis extracts could suppress the serum levels of OVA-specific IgE and IgG_1_, and airway hyperresponsiveness in OVA-sensitized mice. Besides, interferon (IFN)-γ, IL-6, and IL-10 secretion in OVA-stimulated splenocytes from the propolis groups was significantly lower than that of the control group [10].

The composition of propolis is highly variable, depending on the local plant, and is reported to contain approximately 50% resin and vegetable balsam, 30% wax, 10% essential and aromatic oils, 5% pollen, and 5% other substances (minerals) [8]. Further, propolis contains a mixture of biologically active chemicals including terpenes, cinnamic acid, caffeic acid and their esters, amino acids and flavonoids [11]. Caffeic acid phenethyl ester (CAPE), one of the most extensively studied components in propolis, is reported to have anti-tumor [12,13], anti-inflammatory [14,15] and antioxidant [16] properties. CAPE has also been found to suppress eicosanoid synthesis [14]. In immunological studies, CAPE is a potent inhibitor of mitogen-induced T cell proliferation, lymphokine production [17] and nuclear factor (NF)-κB activation [18-20]. CAPE modulated nuclear binding of the NF-κB subunit p65/RelA, decreased expression of cytosolic IκBα [19] and inhibited NFAT dephosphorylation and transcriptional activity [20] in T cells.

Dendritic cells (DCs), one of the most potent professional antigen-presenting cells (APCs), play an important role in the pathogenesis of asthma and allergic rhinitis [21]. DCs normally reside in the airway mucosa and interstitium in an immature state [22] and are specialized in capturing and processing antigens to form major histocompatibility complex (MHC) peptide complexes. Upon antigen or other stimulation, DCs mature with loss of endocytic/phagocytic receptors, upregulation of MHC molecules and costimulatory molecules, alterations in adhesion molecule and cytokine receptor expression and cytokine production, and changes in morphology [23-27]. At the same time, the DCs migrate to the draining lymph nodes and present captured antigen to naïve T lymphocytes [23] by a stable, long-lasting immunologic synapse with T cells. MHC peptide interacts with the T cell receptors, costimulatory molecules interact with T cell-expressed coreceptors, and cytokines are released to polarize the T-cell response [28], thus results in the allergic airway inflammation. The role of DCs in the secondary immune response is further supported by the fact that their depletion at the time of allergen challenge abrogated all the features of asthma in murine model of asthma, including airway inflammation, goblet cell hyperplasia, and bronchial hyperresponsiveness [29].

Since Khayyal *et al*. demonstrated the beneficial effect of propolis in asthma patients [9] and Sy *et al*. reported the similar effect in murine model of asthma [10], we became interested in studying the impact of CAPE on dendritic cells in the pathogenesis of asthma. There was only one study that surveyed the immunosuppressive activity of CAPE on human DCs, which showed that CAPE blocked the *L. major*-induced IRF-1, IRF-8, IL-12 p35 and IL-12 p40 RNA expression in human MoDCs by quantitative real time RT-PCR as well as the IRF-8 nuclear translocation [30]. However, mite and pollens, instead of *L. major*, are the major allergens in allergic disease. In the present study, the authors investigated whether CAPE has regulatory effect on the cytokine and chemokine production and antigen presentation ability in mite stimulated human MoDCs. It is anticipated that this study would establish the role of CAPE on the function of DCs and might provide insight into the mechanisms of action and rationale of propolis administration in the management of asthma and other allergic disorders.

## Results

### CAPE inhibited cytokine and chemokine production in humanMoDCs

**Figure 1:**
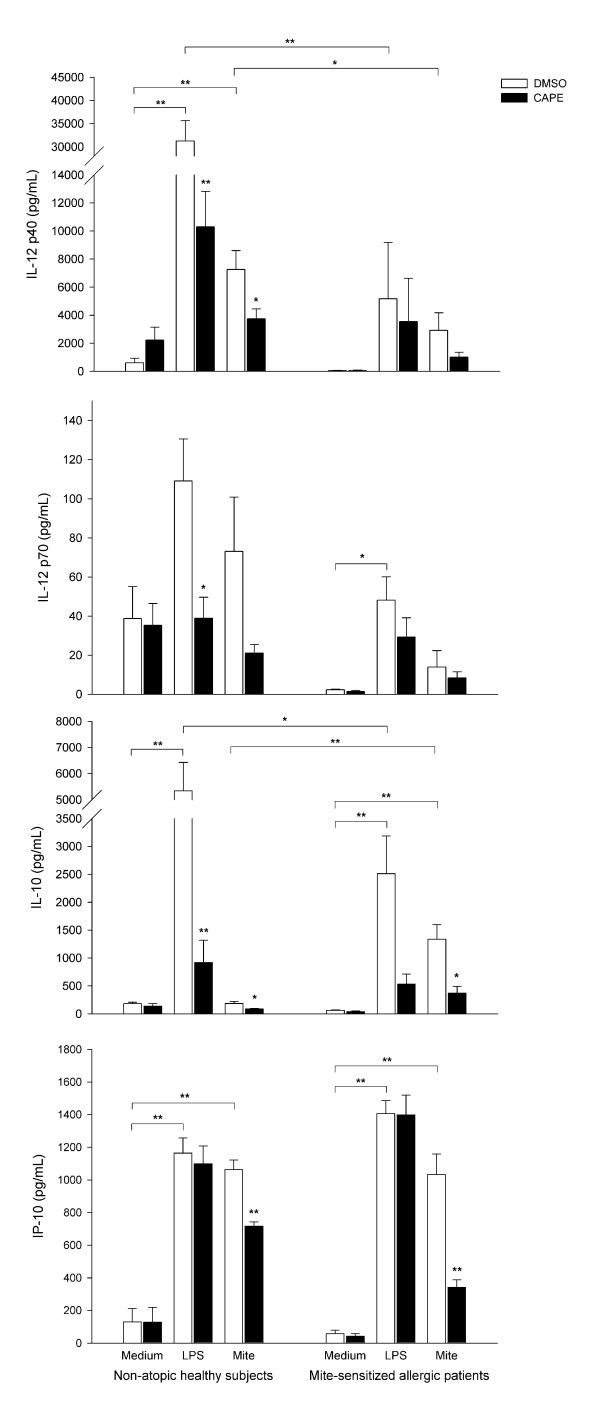
**CAPE inhibited IL-12 p40, IL-12 p70, IL-10 and IP-10 production in human MoDCs**. Immature MoDCs (10^6 ^cells/mL) were cultured for 48 hours either in the absence or presence of CAPE (10 μM) under LPS (100 ng/mL) or crude mite extract (100 μg/mL) stimulation. Cytokine levels in the supernatant from ten non-atopic healthy subjects and six mite-sensitized allergic patients were measured by ELISA according to the manufacturer's instructions. *P < 0.05; **P < 0.01.

The IL-12 p40, IL-12 p70, IL-10 and IFN-γ-inducible protein (IP)-10 levels in the supernatants from non-atopic healthy subjects and mite-sensitized allergic patients were analyzed by ELISA (Figure 1). In healthy subjects, LPS stimulated MoDCs to secrete large amount of IL-12 p40, IL-10 and IP-10 (*P *< 0.001) compared to control MoDCs and crude mite extract stimulated MoDCs to secrete large amount of IL-12 p40 and IP-10 (*P *< 0.001). In allergic patients, LPS stimulated MoDCs to secrete higher IL-12 p70, IL-10 and IP-10 (*P *= 0.018, 0.002, < 0.001, respectively), and crude mite extract stimulated MoDCs to secrete higher IL-10 and IP-10 (*P *< 0.001) than control MoDCs. However, LPS stimulated DCs from allergic patients to secrete less IL-12 p40 and IL-10 (*P *= 0.002, 0.037, respectively) compared to those from healthy subjects. Crude mite extract stimulated DCs from allergic patients to secrete less IL-12 p40 (*P *= 0.046) but higher IL-10 (*P *= 0.003) than those from healthy subjects.

To determine whether CAPE could alter cytokine and chemokine production in human MoDCs, cytokine and chemokine levels of MoDCs cultured for 48 hours under LPS or crude mite extract stimulation in the absence or presence of CAPE were compared. In healthy subjects, CAPE significantly inhibited the IL-12 p40, IL-12 p70 and IL-10 production stimulated by LPS (*P *= 0.001, 0.011, 0.003, respectively). Further, CAPE significantly inhibited the IL-12 p40, IL-10 and IP-10 production stimulated by crude mite extract (*P *= 0.044, 0.022, < 0.001 respectively). IL-12 p70 production stimulated by crude mite extract was also inhibited by CAPE, but this result was not statistically significant (*P *= 0.088). In allergic patients, CAPE could only significantly inhibit the IL-10 and IP-10 production from patients' MoDCs stimulated by crude mite extract (*P *= 0.030, 0.002 respectively).

### CAPE did not alter the upregulation of surface markers on mature MoDCs

**Figure 2:**
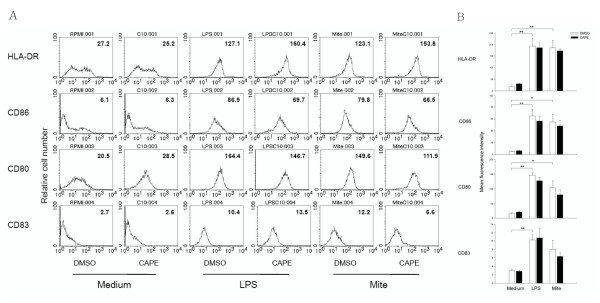
**CAPE did not inhibit costimulatory molecule (such as CD86, CD80, and CD83) expression on MoDCs**. Immature MoDCs from healthy subjects were cultured for 48 hours either in the absence or presence of CAPE (10 μM) under LPS (100 ng/mL) or crude mite extract (100 μg/mL) stimulation. Surface markers were analyzed by flow cytometry. (A) Histogram of fluorescence intensity of HLA-DR, CD86, CD80 and CD83. The values shown in the flow cytometry profiles are the mean fluorescence intensity (MFI) indexes. (B) MFI of HLA-DR, CD86, CD80, and CD83. Data shown represent mean ± SEM from three separate experiments. *P < 0.05; **P < 0.01.

To determine whether CAPE could modulate the maturation of human MoDCs *in vitro*, the phenotype of MoDCs from healthy subjects treated as described above was assessed via analysis of surface markers by flow cytometry (Figure 2). Compared to control MoDCs, MoDCs treated with LPS had increased expression of HLA-DR, CD86, CD80 and CD83 (*P *= 0.007, 0.005, <0.001 and 0.006 respectively) and MoDCs treated with crude mite extract had increased expression of HLA-DR, CD86 and CD80 (*P *= 0.007, 0.018 and 0.020, respectively). In contrast, the costimulatory molecules (CD86, CD80, CD83) and MHC class II molecules were not significantly inhibited by CAPE treatment.

### CAPE did not alter the antigen uptake ability of mature MoDCs

**Figure 3:**
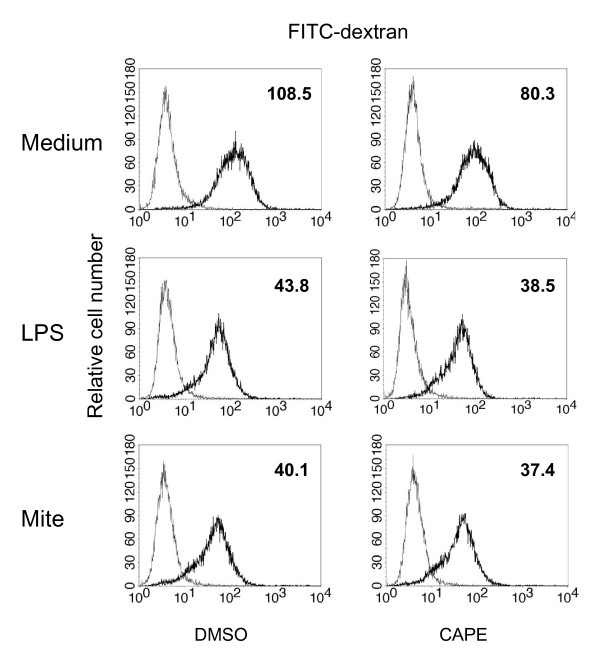
**The effect of CAPE on the phagocytic capacity of human MoDCs**. Immature MoDCs from healthy subjects were cultured for 24 hours either in the absence or presence of CAPE (10 μM) under LPS (100 ng/mL) or crude mite extract (100 μg/mL) stimulation. Cells were then incubated with FITC-dextran for 1 hour at 4°C (thin lines) or 37°C (thick lines). The values shown in the flow cytometry profiles are the MFI indexes. One representative of three independent experiments is shown.

Immature DCs capture antigens through phagocytosis, macropinocytosis and adsorptive endocytosis. Then they become mature DCs and lose their ability of antigen uptake [31]. The uptake of FITC-dextran is known to be maximal in the immature human MoDCs through macropinocytosis and mannose receptor [32]. To determine whether CAPE could modulate the antigen uptake ability of human MoDCs, FITC-dextran was analyzed by flow cytometry (Figure 3). In healthy subjects, the LPS and crude mite extract-treated MoDCs had decreased ability of FITC-dextran uptake compared to control MoDCs. However, CAPE did not alter the phagocytic capacity in human MoDCs.

### CAPE did not alter the T cell activation co-cultured with LPS or mite-treated human MoDCs

**Figure 4:**
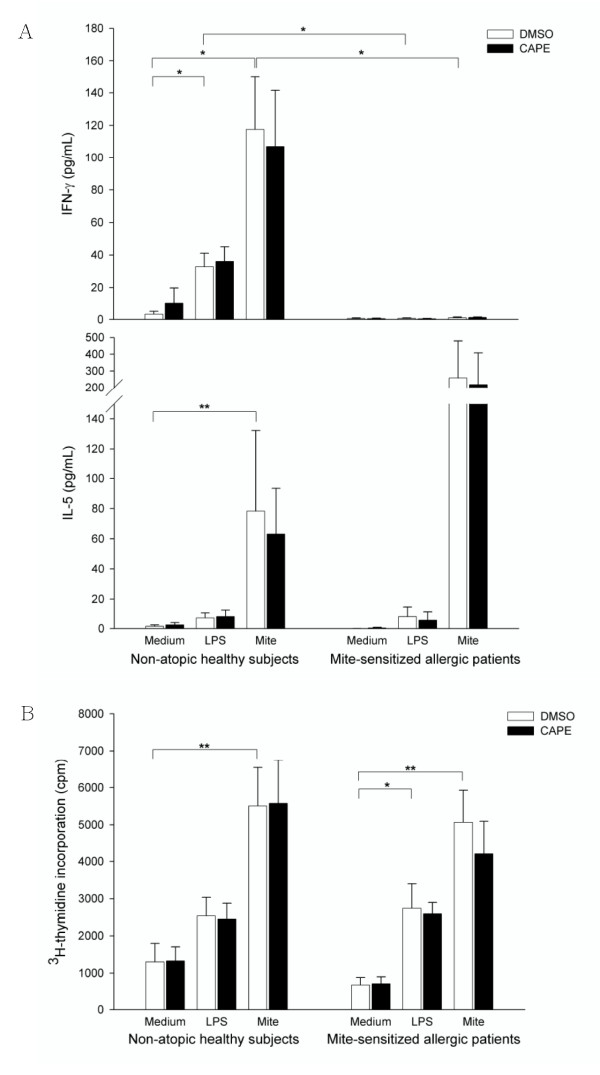
**The effect of CAPE-treated MoDCs on T cell activation**. Mature MoDCs (10^5 ^cells/mL) from eight non-atopic healthy subjects and four mite-sensitized allergic patients treated as indicated were co-cultured with autologous naïve CD4^+ ^T cells (10^6 ^cells/mL). (A) Supernatants were analyzed for IFN-γ and IL-5, which were produced by activated T cells after 3 days of culture. (B) Tritiated(^3^H) thymidine incorporation was measured by liquid scintillation counting after 5 days of culture and expressed as mean counts per minute (cpm). *P < 0.05; **P < 0.01.

Mature DCs have the capacity to induce proliferation and cytokine production in T cells [27]. In the present study, autologous naïve CD4^+ ^T cells was employed in an activation assay to determine if CAPE treated MoDCs have a decreased ability to activate T cells. The treated MoDCs were co-cultured with autologous naïve CD4^+ ^T cells for 3 days in the ratio of 1:10. LPS-pulsed MoDCs induced T cells to secrete IFN-γ compared to controls MoDCs (*P *= 0.023) in healthy subjects (Figure 4A). Crude mite extract-pulsed MoDCs induced T cells to secrete large amount of IFN-γ and IL-5 (*P *= 0.013, < 0.001, respectively) and higher lymphoproliferation (*P *= 0.002) compared to control MoDCs (Figure 4B). In allergic patients, LPS-pulsed MoDCs and crude mite extract-pulsed MoDCs induced higher T cell lymphoproliferation than control MoDCs (*P *= 0.039, 0.008, respectively). IFN-γ secretion by T cells co-cultured with LPS or crude mite extract stimulated MoDCs was higher in healthy subjects than in allergic patients (P = 0.015, 0.012, respectively). There was no difference in T cell cytokine production (IFN-γ and IL-5) and lymphoproliferation between MoDCs that were or were not treated with CAPE in both healthy subjects and allergic patients (Figure 4A and 4B).

### CAPE inhibited IκBα phosphorylation in human MoDCs

**Figure 5:**
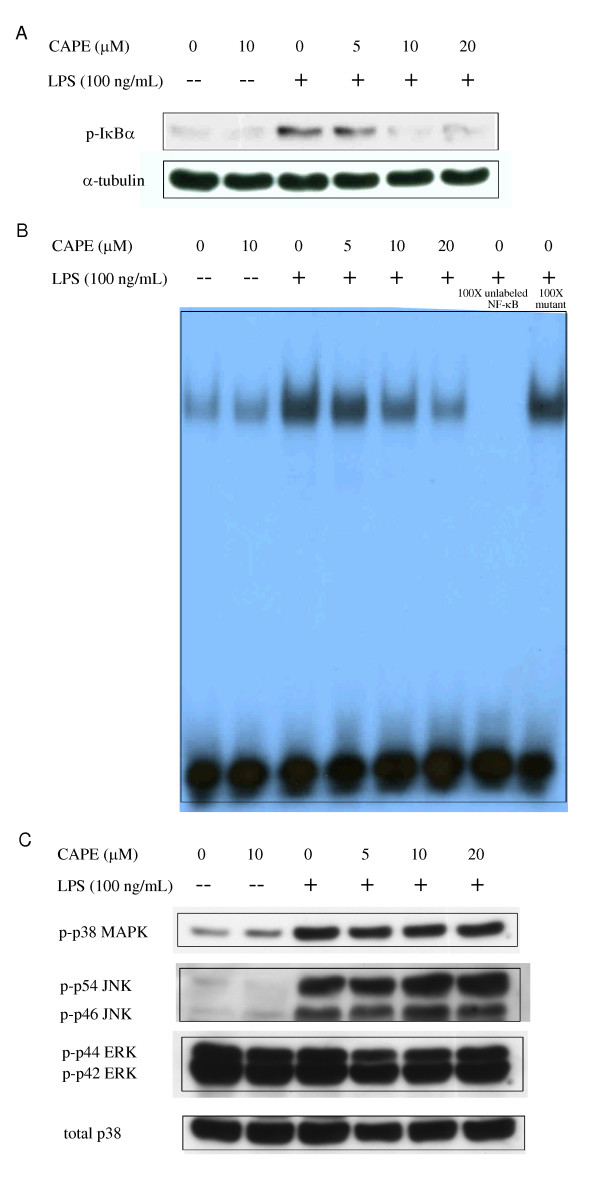
**The effect of CAPE on the NF-κB and MAPK signaling pathway**. Human MoDCs from healthy subjects were pretreated with CAPE in the indicated concentration for 2 hours, then stimulated by LPS (100 ng/mL) for 45 minutes. Cell lysate were collected and the levels of phosphorylation of (A) IκBα and (C) MAPK family (p38 MAPK, p42/44 ERK, and p46/54 JNK kinase) were assayed by Western blotting with indicated Abs. Anti-α-tubulin mAb and anti-total p38 polyclonal Ab were for internal control. (B) The pretreated MoDCs as mentioned were stimulated by LPS (100 ng/mL) for 2 hours and nuclear fractions were prepared and analyzed for NF-κB binding activity by EMSA. To assess the specificity of the binding, 100-fold excess of cold NF-κB probe or mutant probe was added to the LPS condition. One representative of at least three independent experiments is shown.

Since LPS can induce DC maturation through NF-κB activation [33], we used LPS to activate the NF-κB pathway and determine if CAPE could inhibit NF-κB activation in MoDCs. IκBα phosphorylation, related to NF-κB activation, was assessed by Western blot. After pretreating MoDCs with CAPE for 2 hours, LPS was added for 45 minutes to induce NF-κB activation. It was found that CAPE inhibited cytosolic IκBα phosphorylation in a dose-dependent manner (Figure 5A). CAPE could also inhibit the IκBα degradation (see Additional file 1: Figure S1B).

### CAPE inhibited NF-κB activation in human MoDCs

To monitor the inhibitory ability of CAPE on NF-κB translocation into the nucleus, MoDCs pretreated as mentioned were cultured with LPS for 2 hours. The nuclear extracts were analyzed for NF-κB DNA binding by the EMSA. It was found that CAPE inhibited NF-κB translocation and activation in a dose-dependent manner (Figure 5B). The binding of NF-κB was specific and could be blocked by unlabeled, competing NF-κB oligonucleotide.

### CAPE did not inhibit the phosphorylation of MAPK families in human MoDCs

Phosphorylation of p38 mitogen-activated protein kinase (MAPK), p42/44 extracellular signal-regulated kinases (ERK) and P46/54 c-Jun N-terminal kinases (JNK) representing 3 different MAPK activation pathways were evaluated. The levels of MAPK phosphorylation in MoDCs treated as described above (LPS stimulation for 45 minutes) were analyzed by Western blot analysis. It was found that CAPE did not inhibit the phosphorylation of p38 MAPK, p42/44 ERK or P46/54 JNK (Figure 5C).

## Discussion

The main purpose of this study is to elucidate the regulatory effect of CAPE on human MoDCs. First, the cytokine (IL-12 p40, IL-12 p70, and IL-10) and chemokine (IP-10) production from MoDCs were evaluated. In our study, we used LPS as the stimulation other than crude mite extract because LPS is known to be ubiquitously present in the environment and induces Th1- and Th2-related cytokine and chemokine expression [34]. We found that LPS could induce MoDCs to secrete high levels of IL-12, IL-10 and IP-10 in healthy subjects and allergic patients (Figure 1) as previously stated [32,35,36]. Crude mite extract could also stimulate MoDCs to secrete high levels of IL-12 p40 and IP-10 in healthy subjects but high levels of IL-10 and IP-10 in allergic patients. The CAPE treatment could inhibit IL-12 and IL-10 secretion under LPS or crude mite extract stimulation in healthy subjects but only IL-10 secretion under crude mite extract stimulation in allergic patients. CAPE could only inhibit IP-10 secretion under crude mite extract stimulation but not LPS in both healthy subjects and allergic patients. Because the large variation was noted between individuals and the inhibitory effect of CAPE was dose-dependent (data not shown), we speculated that the higher concentration of CAPE may lead to significant IL-12 inhibition in allergic patients.

IL-12, produced by mature DCs, has a central role in initiating a specific T cell-mediated immune response [37,38], driving Th1 cell activation and differentiation [39-42], and inducing production of IFN-γ and lytic activity [43,44] IL-10, the well known regulatory cytokine, can have different functions in immune differentiation and regulation. On one hand, IL-10 and TGF-β have been shown to induce regulatory T cells. These two cytokines, produced early in the infection could activate T cell subsets such as IL-10 and IFN-γ producing T cells [45]. Neutralization of IL-12 inhibited the generation of the double producing T cell lines, indicating the role of IL-12 in inducing IL-10 and IFN-γ producing T cells. Therefore, the principle source of IL-10 in persistent infection may be T cells that also produce IFN-γ, which was important in the modulation of the inflammatory response [45]. On the other hand, IL-10 produced by DCs and macrophages promotes Th2 responses by abrogation of IL-12 generation [46]. DCs from IL-10 treated cultures can induce naïve T cells to differentiate into IL-4-secreting T cells [47], while DCs treated with anti-IL-10 Ab have an increased capacity to activate allogeneic T cells and prime naïve T cells to a more prominent Th1 polarization [48]. Further, the IL-10-induced anergic state in human CD4^+ ^T cells can be reversed by mature DCs [49]. Therefore, the IL-10 produced by DCs may promote Th2 responses but not T cell anergy when co-cultured with mature DCs. In the present study, it appeared that CAPE inhibited cytokine production by MoDCs without preference of Th1 or Th2 prone cytokines in both healthy subjects and allergic patients. IP-10 is a potent chemokine for activated T cells, natural killer cells, and mast cells [50]. In individuals with established allergic inflammation, IP-10 is capable of worsening preexisting asthmatic airway inflammation [51]. CAPE inhibited IP-10 production by crude mite extract-stimulated MoDCs, which could prevent the IP-10 induced airway inflammation in asthmatic patients under mite exposure. Therefore, CAPE inhibited cytokine and chemokine production by MoDCs, and the inhibition was extensive and may have influenced the T cell priming, activation, inflammatory cells recruitment and the further airway inflammation.

NF-κB activation may be responsible, in part, for increased expression of many inflammatory genes in asthma [52]. NF-κB normally binds to IκBα to inhibit NF-κB nuclear translocation from the cytosol to the nucleus for further enhancement of inflammatory and associated gene transcription. Once cells are exposed to inflammatory stimuli such as LPS, IκBα is phosphorylated by IKK complex and degraded, leading to the nuclear translocation of activated NF-κB [53-56]. Different inhibitory results of CAPE on NF-κB signaling pathway were noted in the literatures. In T cells, the direct inhibition of NF-κB nuclear translocation [18,20], and the indirect inhibition of IκBα phosphorylation [20] were noted. In these two articles, CAPE could inhibit IκBα resynthesis instead of cytosolic IκBα degradation [18,20]. However, the inhibitory effect of IκBα degradation of CAPE was observed in human middle ear epithelial cells [57] and gastric epithelial cells [58]. CAPE inhibited IκB degradation in monocytic cells but not astroglial cells, in which the suppression of activated IKK was shown [59]. It seemed that in different cells and possible different concentrations of CAPE (10 μM ~100 μM) would result in different inhibitory mechanisms. In the present study, we found that CAPE inhibited IκBα phosphorylation (Figure 5A) and NF-κB activation (Figure 5B) in human MoDCs in a dose-dependent manner. CAPE could also inhibit IκBα degradation (see Additional file 1 Figure S1B). We speculate that the inhibition of IκBα phosphorylation leads to the inhibition of IκBα degradation, so the nuclear translocation of NF-κB is inhibited for further enhancement of inflammatory and associated gene transcription. Jayakumar *et al*. also reported that pretreatment with CAPE blocked NF-κB p50, NF-κB p65 nuclear translocation in human MoDCs [30]. Since the promoters of hIL-12 p35 and hIL-12 p40 gene contain κB binding sites [60], and previous studies implicates the specific role of NF-κB factors in IL-12 transcription [30,61,62], we also suggested that the inhibition of NF-κB signaling by CAPE might result in the decreased production of IL-12 p40 and p70. The MAPK signaling in the MoDCs maturation may also induce the inflammatory cytokine production [63]. Here, we found that CAPE did not appear to alter the MAPK signaling pathway in human MoDCs, while p38 MAPK and ERK were activated in C6 glioma cells by the treatment of CAPE [64]. Therefore, new experiments addressing the detailed mechanism of inhibition effect of CAPE in cytokine and chemokine production are required.

Upon maturation, DCs can present captured antigens to T cells to induce proliferation and cytokine production in T cells [27]. To mimic in *vivo *system, we chose "autologous" naïve CD4^+ ^T cells for coculture to evaluate the ability of antigen presentation and T cell priming of MoDCs. Surprisingly, CAPE did not inhibit the cytokine production of T cells (Figure 4A) as seen in MoDCs. The lymphoproliferation assay (a marker of T cell activation triggered by APCs) [65] was not inhibited in both healthy subjects and allergic patients when co-cultured with CAPE treated DCs either (Figure 4B). This result might be explained by the fact that the coculture system was in a "renewed" culture medium (2% human AB serum of RPMI). Therefore, the effect of decreased cytokine and chemokine production by MoDCs on T cell priming would be abolished in the experiment. As CAPE did not inhibit the upregulation of MHC molecules and costimulatory molecules in mature MoDCs, the antigen presenting ability of MoDCs in the present study were not expected to differ.

It is interesting about the polarization of crude mite extract. It induced MoDCs to produce large amount of IL-12 p40 but little IL-10 in healthy subjects, and lower IL-12 p40 but higher IL-10 in allergic patients, which indicated that crude mite extract was Th2-prone in allergic patients but not in non-atopic healthy subjects. The phenomenon was also proved in cocultured naïve CD4^+ ^T cells. The lower IFN-γ/IL-5 in allergic patients indicated a Th2 polarization while the higher IFN-γ/IL-5 in healthy subjects favored a Th1 polarization. The different polarizations by Der p-pulsed MoDCs due to the allergic status of the donors were also reported previously [35,36]. These studies used purified Der p 1 for stimulation. They all found that IL-4 or IL-5 was increased in co-cultured autologous CD4^+ ^T cells from mite-sensitized allergic patients while IFN-γ was increased in those from healthy subjects.

The major limitation of our present study is failure of mimicking microenvironment of DC-T cell interaction *in vivo *with cytokine and chemokine in which immunity can develop [66]. Therefore, the effect of the decreased IL-12, IL-10 and IP-10 production by MoDCs treated with CAPE in priming T cells cannot be surveyed in the present study. IL-12 is related to the T cell polarizing and T cell survival [67], thus the decreased IL-12 in microenvironment may influence the T cell activation and proliferation. To determine whether the decreased cytokine secretion of MoDCs would influence the T cells activation, we collected the supernatant of pulsed MoDCs mixed with serum free RPMI 1640 (1:1) to culture autologous naïve CD4^+ ^T cells. The cytokine production and lymphoproliferation were inhibited in CAPE treated group (data not shown). However, the CAPE was in the supernatant and could directly inhibit the T cells activation through NF-κB and NFAT pathway [19,20]. The decreased activation of T cells may be due to both the decreased cytokine and chemokine secretion from MoDCs and the CAPE direct effect. The other limitation is that the *in vitro *human MoDCs cannot represent the *in vivo *tissue DCs. To study the effect of CAPE on the function of circulating DCs will be the future work.

## Conclusion

Our results showed that CAPE significantly inhibited IL-12 p40, IL-12 p70, IL-10 protein expression in mature healthy human MoDCs stimulated by lipopolysaccharides (LPS) and IL-12 p40, IL-10, IP-10 stimulated by crude mite extract. CAPE significantly inhibited IL-10 and IP-10 but not IL-12 expression in allergic patients' MoDCs stimulated by crude mite extract. In contrast, the upregulation of costimulatory molecules in mature MoDCs was not suppressed by CAPE. Therefore, the antigen presentation ability of MoDCs through MHC peptide complex and costimulatory molecules was not changed in this study after evaluating T cell lymphoproliferation and cytokine production. The mechanism of the inhibition of cytokine and chemokine production in MoDCs was thought to be related to NF-κB signaling, but not the MAPK pathway. The findings of this study provide new information and evidence for regulatory effect of CAPE on MoDCs relevant to the pathogenesis of allergic airway disease, such as asthma and allergic rhinitis. Further, it is possible to suggest that CAPE and propolis might be useful in the management of allergic disorders since CAPE inhibited cytokine and chemokine production in MoDCs and thus resulted in a decrease in downstream inflammatory process.

## Methods

### Human subjects

The mite-sensitized allergic patients [three had allergic rhinitis, three had asthma, and all had 4+ of mite-specific IgE (MAST, HITACHI CLA test)] and non-atopic healthy subjects were enrolled for peripheral blood sampling. This study was approved by the Research Ethics Committee of the National Taiwan University Hospital and informed consent was obtained from all subjects.

### Reagents

CAPE and Escherichia coli LPS (L8274, E. coli) were purchased from Sigma-Aldrich Chemical Co. (St. Louis, MO). CAPE was dissolved in DMSO. Cells without CAPE treatment were treated with DMSO only (0.1% of culture medium, v/v). Isotopes were obtained from Amersham Corp. (Arlington Heights, IL).

### Crude mite extract from *Dermatophagoides pteronyssinus*

Lyophilized house dust mite (*Dermatophagoides pteronyssinus) *was purchased from Allergon (Angelholm, Sweden). The allergen was prepared as described previously [68]. Briefly, 1 g of lyophilized mite body was defatted with 100 mL ether, homogenized and stirred continuously in 25 mL phosphate-buffered saline (PBS) for 48 hours at 4°C. After centrifugation (12000 *g *for 30 minutes), the crude mite extract was dialysed with PBS and then dissolved in PBS and stored at -20°C.

### Isolation of human peripheral monocytes and generation of human MoDCs

MoDCs were generated from peripheral monocytes, as described previously [32]. Peripheral mononuclear cells (PBMCs) were isolated from heparinized peripheral blood by Ficoll-Hypaque (GE Healthcare, Buckinghamshire, UK) density gradient centrifugation (400 *g *for 30 minutes at room temperature). The mononuclear cells layer was washed twice with sterile Hank's solution (Sigma-Aldrich). CD14^+ ^cells were purified by positive selection using anti-CD14^+ ^conjugated magnetic microbeads by autoMACS according to the manufacturer's protocol (Miltenyi Biotec, Auburn, CA). The CD14^+ ^monocytes were then cultured in RPMI 1640 medium (SAFC Biosciences, Lenexa, Kansas) containing 10% fetal calf serum (FCS), 2 mM L-glutamine, 100 U/mL penicillin, 100 U/mL streptomycin, and 25 mM HEPES with granulocyte macrophage-colony stimulating factor (GM-CSF; 800 U/mL) and IL-4 (200 U/mL) in 24-well plates (Costar, Cambridge, MA) plated at a concentration of 10^6 ^cells/mL for 5 days (37°C/5% CO_2_). Human MoDCs were routinely used at day 6 of culture. Immature MoDCs were treated with LPS (100 ng/mL) or crude mite extract (100 μg/mL) in the absence or presence of CAPE (10 μM) for an additional 2 days to obtain mature DCs. The doses and time-points chosen were according to the literature [20,32] and further confirmed in the pilot experiments (data not shown).

### Co-culture of MoDCs and autologous T cells

PBMCs were obtained as described above and naïve CD4^+ ^T cells were purified from PBMCs using a naïve CD4^+ ^T cell isolation kit (Miltenyi Biotec) according to the manufacturer's protocol. Autologous naïve CD4^+ ^T cells were cultured with RPMI 1640 with 2% human AB serum in the concentration of 10^6 ^cells/mL in 96-well round bottom plates in triplicate for 3 days in combination with mature MoDCs (10^5 ^cells/mL). Tritiated thymidine (1 μCi/well, New England Nuclear, Boston, MA) incorporation for 16 hours was determined with a liquid scintillation counter.

### Determination of cytokine and chemokine levels

Concentrations of IFN-γ, IL-5, IL-10, IL-12 p40, IL-12 p70, and IP-10 in the culture supernatant from MoDCs or T cells were assayed by an enzyme-linked immunosorbent assay (ELISA) kit (R&D Systems, Minneapolis, MN) according to each of the manufacturer's protocol.

### Flow cytometry analysis

MoDCs were harvested and washed with cold buffer (PBS containing 2% FCS and 0.1% sodium azide). Cells were stained with the monoclonal antibodies of CD14, HLA-DR, CD80, CD83 or CD86 (Becton Dickinson, San Jose, CA) or isotype-matched controls for 30 minutes on ice. Stained cells were then washed and resuspended in cold buffer and analyzed with a FACSort cell analyzer (Becton Dickinson). More than 1 × 10^4 ^cells were analyzed for each sample and the results were processed by Cellquest software (Becton Dickinson).

### Phagocytic capacity analysis

MoDCs were washed twice, resuspended in RPMI 1640 medium containing 10% FCS and rest on ice for 30 minutes. The cells were then incubated with FITC-labeled dextran (0.2 mg/mL; Invitrogen, Carlsbad, CA) at 4°C or 37°C for 1 hour. Finally, the cells were washed thrice with cold buffer and analyzed with a FACSort cell analyzer, as described above.

### Western blotting

Human MoDCs were pre-treated with CAPE (0, 5, 10 or 20 μM) for 2 hours. LPS (100 ng/mL) was then added for 45 minutes (37°C/5% CO_2_), then the total cellular extract was prepared using Gold lysis buffer [10% glycerol, 1% Triton X-100, 1 mM sodium orthovanadate, 1 mM EGTA, 5 mM EDTA, 10 mM NaF, 1 mM sodium pyrophosphate, 20 mM Tris-HCl, pH 7.9, 100 μM β-glycerophosphate, 137 mM NaCl, 1 mM phenylmethylsulfonyl fluoride (PMSF), 10 μg/mL aprotinin, and 10 μg/mL leupeptin] for 30 minutes at 4°C. The cell lysate was clarified by centrifugation at 12,000 *g *for 15 minutes at 4°C. Total protein (35 μg) was separated on 10% sodium dodecyl sulfate (SDS)-polyacrylamide minigels and transferred to Immobilon polyvinylidene difluoride membrane (Millipore, Bedford, MA). The membrane was incubated for 1 hour at room temperature with 5% nonfat dry milk in PBS to block nonspecific immunoglobulins, then incubated with anti-α-tubulin mAb (Santa Cruz Biotechnology, Santa Cruz, California), anti-IκBα, anti-IκBα-P, anti-p38-P, anti-p42/44-P, anti-p46/54-P, or anti-total p38 polyclonal antibodies (Cell Signaling Technology, Beverly, MA). Immunodetection was carried out by an Amersham enhanced chemiluminescence plus system (GE Healthcare). The time-points chosen were according to the literature [20,32] and further confirmed in the pilot experiments (see Additional file 1: Figure S1).

### Preparation of nuclear extracts and electrophoretic mobility shift assay (EMSA)

Nuclear extracts were prepared by NE-PER nuclear and cytoplasmic extraction reagent kit (Pierce Biotechnology, Rockford, IL) according to the manufacturer's protocol. Each 5 μg nuclear extract was mixed with the labeled double-stranded NF-κB oligonucleotide, 5'-AGTTGA**GGGGACTTTCCC**AGGC-3', and incubated at room temperature for 20 min. The incubation mixture included 1 μg of poly (dI-dC) in a binding buffer (25 mM HEPES, pH 7.9, 0.5 mM EDTA, 0.5 mM DTT, 1% Nonidet P-40, 5% glycerol, and 50 mM NaCl). The DNA-protein complex was electrophoresed on 4.5% non-denaturing polyacrylamide gels in 0.5× TBE buffer (0.0445 M Tris, 0.0445 M borate, 0.001 M EDTA). A double-stranded mutated oligonucleotide, 5'-AGTTGA**GGCGACTTTCCC**AGGC-3', was used to examine the specificity of the binding of NF-κB to DNA. The specificity of binding was also examined by comparison with the unlabelled oligonucleotide.

### Statistics

Raw data were expressed as mean ± standard error of mean (SEM) and were analyzed by the Student's *t *test to determine statistical differences between experimental groups. *P*-values lower than 0.05 were considered to be significant.

## Abbreviations

CAPE: caffeic acid phenethyl ester; DCs: dendritic cells; ERK: extracellular signal-regulated kinases; IFN-γ: interferon-γ; IP-10: IFN-γ-inducible protein 10; IKK: IκB kinase; IL: interleukin; JNK: c-Jun N-terminal kinases; LPS: lipopolysaccharides; MAPK: mitogen-activated protein kinase; NF-κB: nuclear factor-κB.

## Authors' contributions

LCW and YLL performed the experimental work, analyses and interpretation of data, drafted the manuscript and were involved in the conception and design of the study. YCL performed the EMSA. BLC was involved with analysis, interpretation of data, drafted the manuscript and made substantial contributions to design of the study. YHY, JHL, HHY, and WMW made substantial contributions to conception and design of the study, and were involved in critically revising the manuscript. All authors read and approved the final manuscript.

## Supplementary Material

Additional file 1 **Figure S1 – LPS induced IκBα phosphorylation and degradation in time kinetics**. Human MoDCs from healthy subjects were pretreated with CAPE (10 μM) for 2 hours, then stimulated by LPS (100 ng/mL) in the indicated time. Cell lysate were collected and the levels of (A) phosphorylated IκBα and (B) IκBα were assayed by Western blotting with indicated Abs. Anti-α-tubulin mAb was for internal control. Click here for file

## References

[B1] Carlsen KH (2005). Pharmaceutical treatment of asthma in children. Curr Drug Targets Inflamm Allergy.

[B2] Nelson HS, Weiss ST, Bleecker ER, Yancey SW, Dorinsky PM (2006). The Salmeterol Multicenter Asthma Research Trial: a comparison of usual pharmacotherapy for asthma or usual pharmacotherapy plus salmeterol. Chest.

[B3] Paul O'Byrne EDB, Bousquet Jean, Clark Tim, Ohta Ken, Paggiaro Pierluigi, Pedersen Soren Erik, Soto-Quiroz Manuel, Singh RajB, Tan Wang-Cheng (2007). Global strategy for asthma management and prevention 2007.

[B4] Canonica GW, Passalacqua G (2003). Noninjection routes for immunotherapy. J Allergy Clin Immunol.

[B5] Sopo SM, Macchiaiolo M, Zorzi G, Tripodi S (2004). Sublingual immunotherapy in asthma and rhinoconjunctivitis; systematic review of paediatric literature. Arch Dis Child.

[B6] Rienzo VD, Minelli M, Musarra A, Sambugaro R, Pecora S, Canonica WG, Passalacqua G (2005). Post-marketing survey on the safety of sublingual immunotherapy in children below the age of 5 years. Clin Exp Allergy.

[B7] Wang LC, Lee JH, Yang YH, Lin YT, Chiang BL (2007). New biological approaches in asthma: DNA-based therapy. Curr Med Chem.

[B8] Cohen HA, Varsano I, Kahan E, Sarrell EM, Uziel Y (2004). Effectiveness of an herbal preparation containing echinacea, propolis, and vitamin C in preventing respiratory tract infections in children: a randomized, double-blind, placebo-controlled, multicenter study. Arch Pediatr Adolesc Med.

[B9] Khayyal MT, el-Ghazaly MA, el-Khatib AS, Hatem AM, de Vries PJ, el-Shafei S, Khattab MM (2003). A clinical pharmacological study of the potential beneficial effects of a propolis food product as an adjuvant in asthmatic patients. Fundam Clin Pharmacol.

[B10] Sy LB, Wu YL, Chiang BL, Wang YH, Wu WM (2006). Propolis extracts exhibit an immunoregulatory activity in an OVA-sensitized airway inflammatory animal model. Int Immunopharmacol.

[B11] Volpert R, Elstner EF (1993). Biochemical activities of propolis extracts. I. Standardization and antioxidative properties of ethanolic and aqueous derivatives. Z Naturforsch [C].

[B12] Chiao C, Carothers AM, Grunberger D, Solomon G, Preston GA, Barrett JC (1995). Apoptosis and altered redox state induced by caffeic acid phenethyl ester (CAPE) in transformed rat fibroblast cells. Cancer Res.

[B13] Huang MT, Ma W, Yen P, Xie JG, Han J, Frenkel K, Grunberger D, Conney AH (1996). Inhibitory effects of caffeic acid phenethyl ester (CAPE) on 12-O-tetradecanoylphorbol-13-acetate-induced tumor promotion in mouse skin and the synthesis of DNA, RNA and protein in HeLa cells. Carcinogenesis.

[B14] Mirzoeva OK, Calder PC (1996). The effect of propolis and its components on eicosanoid production during the inflammatory response. Prostaglandins Leukot Essent Fatty Acids.

[B15] Michaluart P, Masferrer JL, Carothers AM, Subbaramaiah K, Zweifel BS, Koboldt C, Mestre JR, Grunberger D, Sacks PG, Tanabe T (1999). Inhibitory effects of caffeic acid phenethyl ester on the activity and expression of cyclooxygenase-2 in human oral epithelial cells and in a rat model of inflammation. Cancer Res.

[B16] Ahn MR, Kumazawa S, Hamasaka T, Bang KS, Nakayama T (2004). Antioxidant activity and constituents of propolis collected in various areas of Korea. J Agric Food Chem.

[B17] Ansorge S, Reinhold D, Lendeckel U (2003). Propolis and some of its constituents down-regulate DNA synthesis and inflammatory cytokine production but induce TGF-beta1 production of human immune cells. Z Naturforsch [C].

[B18] Natarajan K, Singh S, Burke TR, Grunberger D, Aggarwal BB (1996). Caffeic acid phenethyl ester is a potent and specific inhibitor of activation of nuclear transcription factor NF-kappa B. Proc Natl Acad Sci USA.

[B19] Orban Z, Mitsiades N, Burke TR, Tsokos M, Chrousos GP (2000). Caffeic acid phenethyl ester induces leukocyte apoptosis, modulates nuclear factor-kappa B and suppresses acute inflammation. Neuroimmunomodulation.

[B20] Marquez N, Sancho R, Macho A, Calzado MA, Fiebich BL, Munoz E (2004). Caffeic acid phenethyl ester inhibits T-cell activation by targeting both nuclear factor of activated T-cells and NF-kappaB transcription factors. J Pharmacol Exp Ther.

[B21] van Rijt LS, Lambrecht BN (2005). Dendritic cells in asthma: a function beyond sensitization. Clin Exp Allergy.

[B22] Lambrecht BN, van Rijt LS (2006). Infections and asthma pathogenesis: a critical role for dendritic cells?. Novartis Found Symp.

[B23] Schuurhuis DH, Fu N, Ossendorp F, Melief CJ (2006). Ins and outs of dendritic cells. Int Arch Allergy Immunol.

[B24] Kleijmeer M, Ramm G, Schuurhuis D, Griffith J, Rescigno M, Ricciardi-Castagnoli P, Rudensky AY, Ossendorp F, Melief CJ, Stoorvogel W (2001). Reorganization of multivesicular bodies regulates MHC class II antigen presentation by dendritic cells. J Cell Biol.

[B25] Turley SJ, Inaba K, Garrett WS, Ebersold M, Unternaehrer J, Steinman RM, Mellman I (2000). Transport of peptide-MHC class II complexes in developing dendritic cells. Science.

[B26] Banchereau J, Briere F, Caux C, Davoust J, Lebecque S, Liu YJ, Pulendran B, Palucka K (2000). Immunobiology of dendritic cells. Annu Rev Immunol.

[B27] Reis e Sousa C (2006). Dendritic cells in a mature age. Nat Rev Immunol.

[B28] Hammad H, Lambrecht BN (2006). Recent progress in the biology of airway dendritic cells and implications for understanding the regulation of asthmatic inflammation. J Allergy Clin Immunol.

[B29] van Rijt LS, Jung S, Kleinjan A, Vos N, Willart M, Duez C, Hoogsteden HC, Lambrecht BN (2005). In vivo depletion of lung CD11c+ dendritic cells during allergen challenge abrogates the characteristic features of asthma. J Exp Med.

[B30] Jayakumar A, Donovan MJ, Tripathi V, Ramalho-Ortigao M, McDowell MA (2008). Leishmania major infection activates NF-kappaB and interferon regulatory factors 1 and 8 in human dendritic cells. Infect Immun.

[B31] Banchereau J, Steinman RM (1998). Dendritic cells and the control of immunity. Nature.

[B32] Lin YL, Liang YC, Lee SS, Chiang BL (2005). Polysaccharide purified from Ganoderma lucidum induced activation and maturation of human monocyte-derived dendritic cells by the NF-kappaB and p38 mitogen-activated protein kinase pathways. J Leukoc Biol.

[B33] Duez C, Gosset P, Tonnel AB (2006). Dendritic cells and toll-like receptors in allergy and asthma. Eur J Dermatol.

[B34] Hung CH, Chu YT, Hua YM, Hsu SH, Lin CS, Chang HC, Lee MS, Jong YJ (2008). Effects of formoterol and salmeterol on the production of Th1- and Th2-related chemokines by monocytes and bronchial epithelial cells. Eur Respir J.

[B35] Hammad H, Charbonnier AS, Duez C, Jacquet A, Stewart GA, Tonnel AB, Pestel J (2001). Th2 polarization by Der p 1–pulsed monocyte-derived dendritic cells is due to the allergic status of the donors. Blood.

[B36] De Wit D, Amraoui Z, Vincart B, Michel O, Michils A, Van Overvelt L, Willems F, Goldman M (2000). Helper T-cell responses elicited by Der p 1-pulsed dendritic cells and recombinant IL-12 in atopic and healthy subjects. J Allergy Clin Immunol.

[B37] Trinchieri G (1998). Interleukin-12: a cytokine at the interface of inflammation and immunity. Adv Immunol.

[B38] Sinigaglia F, D'Ambrosio D, Panina-Bordignon P, Rogge L (1999). Regulation of the IL-12/IL-12R axis: a critical step in T-helper cell differentiation and effector function. Immunol Rev.

[B39] Foti M, Granucci F, Ricciardi-Castagnoli P (2006). Dendritic cell interactions and cytokine production. Ernst Schering Res Found Workshop.

[B40] Hsieh CS, Macatonia SE, Tripp CS, Wolf SF, O'Garra A, Murphy KM (1993). Development of TH1 CD4+ T cells through IL-12 produced by Listeria-induced macrophages. Science.

[B41] Hendrzak JA, Brunda MJ (1995). Interleukin-12. Biologic activity, therapeutic utility, and role in disease. Lab Invest.

[B42] Shikano H, Kato Z, Kaneko H, Watanabe M, Inoue R, Kasahara K, Takemura M, Kondo N (2001). IFN-gamma production in response to IL-18 or IL-12 stimulation by peripheral blood mononuclear cells of atopic patients. Clin Exp Allergy.

[B43] Galon J, Sudarshan C, Ito S, Finbloom D, O'Shea JJ (1999). IL-12 induces IFN regulating factor-1 (IRF-1) gene expression in human NK and T cells. J Immunol.

[B44] Biron CA, Nguyen KB, Pien GC, Cousens LP, Salazar-Mather TP (1999). Natural killer cells in antiviral defense: function and regulation by innate cytokines. Annu Rev Immunol.

[B45] Trinchieri G (2001). Regulatory role of T cells producing both interferon gamma and interleukin 10 in persistent infection. J Exp Med.

[B46] Moore KW, de Waal Malefyt R, Coffman RL, O'Garra A (2001). Interleukin-10 and the interleukin-10 receptor. Annu Rev Immunol.

[B47] Liu L, Rich BE, Inobe J, Chen W, Weiner HL (1998). Induction of Th2 cell differentiation in the primary immune response: dendritic cells isolated from adherent cell culture treated with IL-10 prime naive CD4+ T cells to secrete IL-4. Int Immunol.

[B48] Corinti S, Albanesi C, la Sala A, Pastore S, Girolomoni G (2001). Regulatory activity of autocrine IL-10 on dendritic cell functions. J Immunol.

[B49] Chen ML, Wang FH, Lee PK, Lin CM (2001). Interleukin-10-induced T cell unresponsiveness can be reversed by dendritic cell stimulation. Immunol Lett.

[B50] Luster AD, Unkeless JC, Ravetch JV (1985). Gamma-interferon transcriptionally regulates an early-response gene containing homology to platelet proteins. Nature.

[B51] Wark PA, Bucchieri F, Johnston SL, Gibson PG, Hamilton L, Mimica J, Zummo G, Holgate ST, Attia J, Thakkinstian A (2007). IFN-gamma-induced protein 10 is a novel biomarker of rhinovirus-induced asthma exacerbations. J Allergy Clin Immunol.

[B52] Hart LA, Krishnan VL, Adcock IM, Barnes PJ, Chung KF (1998). Activation and localization of transcription factor, nuclear factor-kappaB, in asthma. Am J Respir Crit Care Med.

[B53] Lindner I, Cejas PJ, Carlson LM, Torruellas J, Plano GV, Lee KP (2007). Signal transduction in DC differentiation: winged messengers and Achilles' heel. Adv Exp Med Biol.

[B54] Baeuerle PA, Henkel T (1994). Function and activation of NF-kappa B in the immune system. Annu Rev Immunol.

[B55] Ghosh S, May MJ, Kopp EB (1998). evolutionarily conserved mediators of immune responses. Annu Rev Immunol.

[B56] Wulczyn FG, Krappmann D, Scheidereit C (1996). The NF-kappa B/Rel and I kappa B gene families: mediators of immune response and inflammation. J Mol Med.

[B57] Song JJ, Cho JG, Hwang SJ, Cho CG, Park SW, Chae SW (2008). Inhibitory effect of caffeic acid phenethyl ester (CAPE) on LPS-induced inflammation of human middle ear epithelial cells. Acta Otolaryngol.

[B58] Abdel-Latif MM, Windle HJ, Homasany BS, Sabra K, Kelleher D (2005). Caffeic acid phenethyl ester modulates Helicobacter pylori-induced nuclear factor-kappa B and activator protein-1 expression in gastric epithelial cells. Br J Pharmacol.

[B59] Choi K, Choi C (2008). Differential regulation of c-Jun N-terminal kinase and NF-kappaB pathway by caffeic acid phenethyl ester in astroglial and monocytic cells. J Neurochem.

[B60] Yoshimoto T, Kojima K, Funakoshi T, Endo Y, Fujita T, Nariuchi H (1996). Molecular cloning and characterization of murine IL-12 genes. J Immunol.

[B61] Kollet J, Witek C, Gentry JD, Liu X, Schwartzbach SD, Petro TM (2001). Deletional analysis of the murine IL-12 p35 promoter comparing IFN-gamma and lipopolysaccharide stimulation. J Immunol.

[B62] Liu J, Cao S, Herman LM, Ma X (2003). Differential regulation of interleukin (IL)-12 p35 and p40 gene expression and interferon (IFN)-gamma-primed IL-12 production by IFN regulatory factor 1. J Exp Med.

[B63] Nakahara T, Moroi Y, Uchi H, Furue M (2006). Differential role of MAPK signaling in human dendritic cell maturation and Th1/Th2 engagement. J Dermatol Sci.

[B64] Lee YJ, Kuo HC, Chu CY, Wang CJ, Lin WC, Tseng TH (2003). Involvement of tumor suppressor protein p53 and p38 MAPK in caffeic acid phenethyl ester-induced apoptosis of C6 glioma cells. Biochem Pharmacol.

[B65] Inaba K, Steinman RM (1984). Resting and sensitized T lymphocytes exhibit distinct stimulatory (antigen-presenting cell) requirements for growth and lymphokine release. J Exp Med.

[B66] Ingulli E, Mondino A, Khoruts A, Jenkins MK (1997). In vivo detection of dendritic cell antigen presentation to CD4(+) T cells. J Exp Med.

[B67] Ruby CE, Montler R, Zheng R, Shu S, Weinberg AD (2008). IL-12 is required for anti-OX40-mediated CD4 T cell survival. J Immunol.

[B68] Lee YL, Fu CL, Ye YL, Chiang BL (1999). Administration of interleukin-12 prevents mite Der p 1 allergen-IgE antibody production and airway eosinophil infiltration in an animal model of airway inflammation. Scand J Immunol.

