# Supplementary figures and images for: The effect of caffeic acid phenethyl ester on the functions of human monocyte-derived dendritic cells
Source: BMC Immunol. 2009 Jul 16;10:39. doi: 10.1186/1471-2172-10-39 (PMC2724478; doi:10.1186/1471-2172-10-39)

A

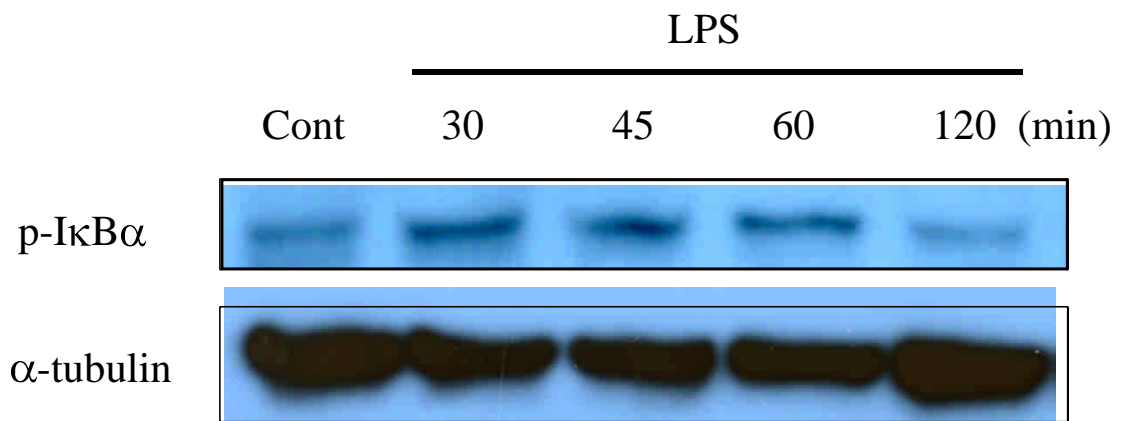

B

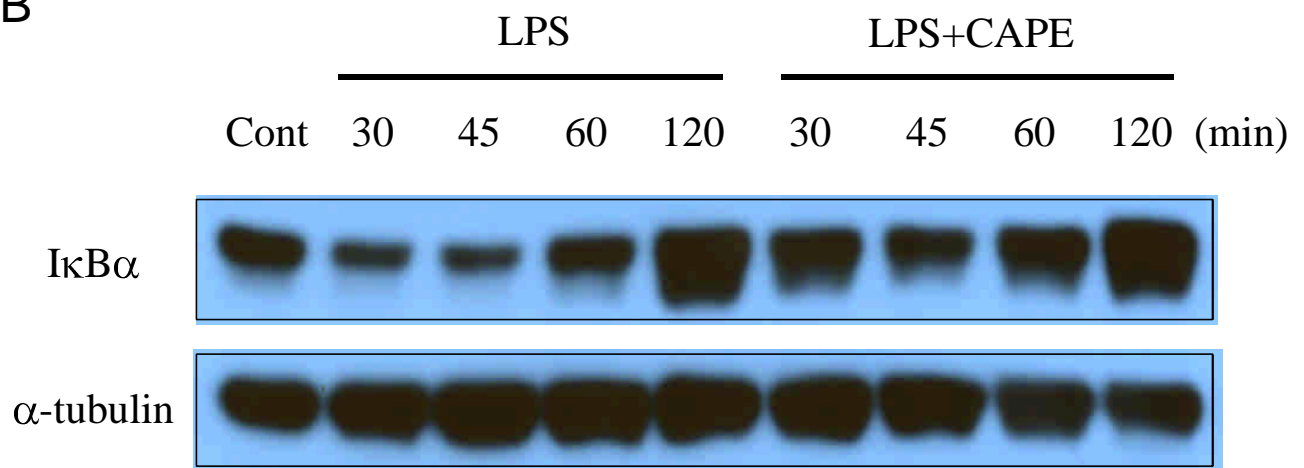

Supplement: Additional file 1 — Figure S1 – LPS induced IκBα phosphorylation and degradation in time kinetics. Human MoDCs from healthy subjects were pretreated with CAPE (10 μM) for 2 hours, then stimulated by LPS (100 ng/mL) in the indicated time. Cell lysate were collected and the levels of (A) phosphorylated IκBα and (B) IκBα were assayed by Western blotting with indicated Abs. Anti-α-tubulin mAb was for internal control. [file 1471-2172-10-39-S1.pdf]
